# GATA1-induced upregulation of LINC01503 promotes carboplatin resistance in ovarian carcinoma by upregulating PD-L1 via sponging miR-766-5p

**DOI:** 10.1186/s13048-021-00856-3

**Published:** 2021-08-23

**Authors:** Yao Li, Yan Zhai, Yuxuan Chen

**Affiliations:** 1 Department of Gynaecology and Obstetrics, Beijing Chao-Yang Hospital, Capital Medical University, No. 8 South Workers Stadium Road, Chaoyang District, Beijing, 100020 China; 2 Department of Gynecology, Capital Medical University, Beijing, 100071 China

**Keywords:** GATA1, LINC01503, Carboplatin, Ovarian Carcinoma, PD-L1, miR-766-5p

## Abstract

**Background:**

Ovarian Carcinoma (OCa) is a high-mortality malignancy derived from female reproductive system. Increasing evidence has identified long non-coding RNAs (lncRNAs) as important regulators in OCa chemoresistance. In this study, we intended to explore the role of LINC01503 in OCa resistance to carboplatin (CBP).

**Methods:**

Gene expression was measured by reverse transcription-quantitative PCR (RT-qPCR) in OCa cells. Western blot was adopted to detect protein levels of GATA1, PD-L1, E-cadherin, N-cadherin, Vimentin, Bcl-2, Bax, cleaved caspase-3. To assess the effects of LINC01503 on the resistance of OCa cells to CBP, Cell Counting Kit-8 (CCK-8), colony formation, Transwell, and flow cytometry experiments were performed to evaluate half-maximal inhibitory concentration (IC_50_), cell viability, migrative and invasive ability, as well as cell apoptosis. Dual-luciferase reporter assay was employed to assess the associations between the genes.

**Results:**

LINC01503 was upregulated in CBP-resistant OCa cells. LINC01503 knockdown reduced CBP resistance in OCa cells. Besides, GATA-binding protein 1 (GATA1) activated LINC01503 transcription in CBP-resistant OCa cells. MiR-766-5p was lowly expressed in CBP-resistant cells and confirmed as a target for LINC01503. In addition, miR-766-5p overexpression increased CBP sensitivity in OCa cells. PD-L1 was verified as the target of miR-766-5p. Besides, LINC01503 upregulated PD-L1 level by regulating miR-766-5p. Furthermore, rescue experiments showed that PD-L1 overexpression abrogated the inhibited impacts of blocking LINC01503 on CBP resistance in OCa cells.

**Conclusion:**

GATA1-induced LINC01503 expedited CBP resistance in OCa cells via the miR-766-5p/PD-L1 axis, providing a new target for improving the efficacy of OCa chemotherapy.

**Graphical Abstract:**

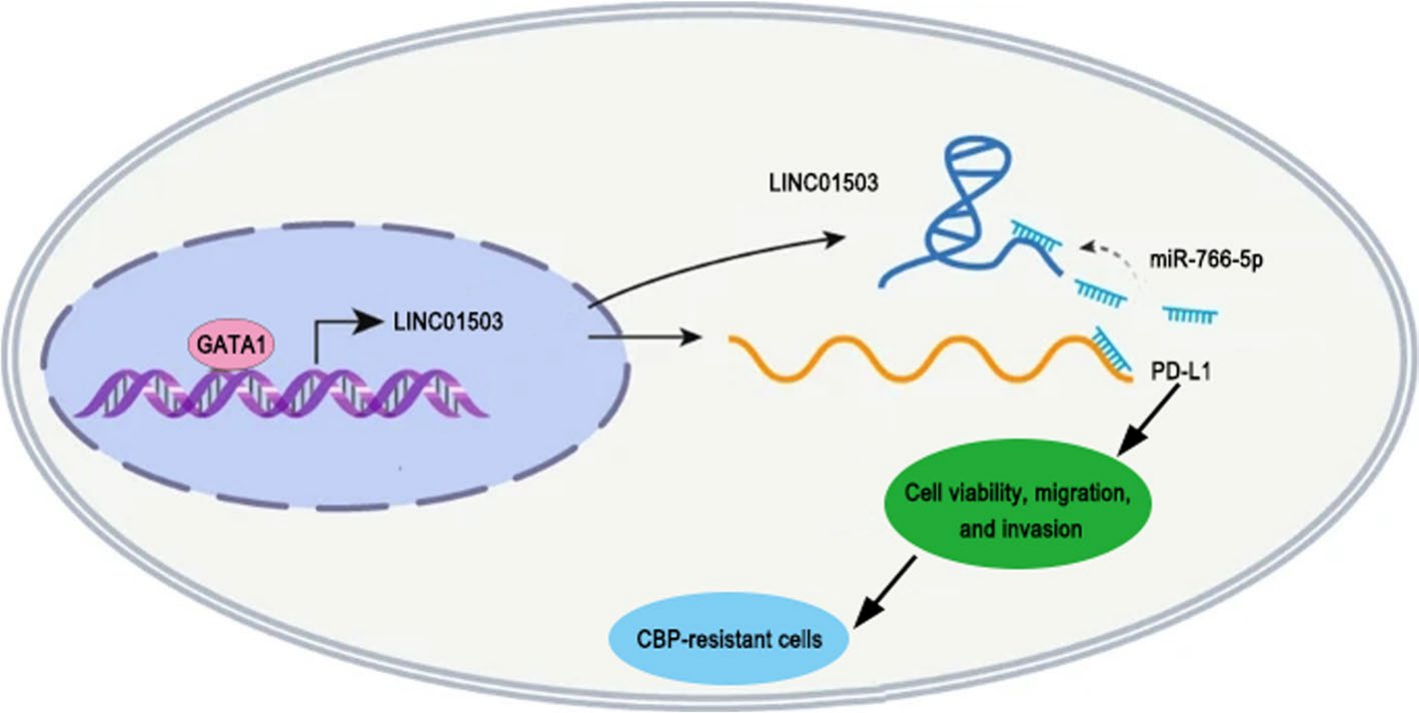

## Introduction

As a major fatal cancer type among gynecologic malignancies worldwide, Ovarian Carcinoma (OCa) is characterized by multidrug resistance, early-onset metastasis, recurrence, and poor prognosis [1, 2]. Due to untypical symptoms and uncertain screening results, a great number of OCa patients were diagnosed at advanced stages, resulting in an unsatisfactory 5-year survival rate of OCa patients [3, 4]. Currently, the first-line therapy for patients with OCa is mainly surgical resection supplemented by chemotherapy with platinum-based drugs, including carboplatin (CBP) [5, 6]. Chemotherapy can effectively prolong the survival time of patients with advanced OCa; however, longtime chemotherapy may also induce drug resistance, thereby leading to recurrence, metastasis, and even death [7]. Hence, a better understanding of the chemoresistance-associated mechanisms in OCa is necessary to improve the prognosis of OCa patients.

Long noncoding RNAs (lncRNAs) are RNAs (> 200 nucleotides) with no protein-coding capabilities [8]. Increasing evidence has demonstrated that multiple lncRNAs are dysregulated in human cancers and participated in tumor progression and even drug resistance. It has been identified by a number of studies that lncRNA can promote OCa progression and chemoresistance. To cite an instance, Li et al. reported that lncRNA UCA1 conferred cisplatin resistance in OCa by upregulating FOSL2 via miR-143 [9]. Zhao et al. discovered that lncRNA SDHAP1 attenuated sensitivity to paclitaxel in OCa via the miR-4465/EIF4G2 axis [10]. A report from Zhao et al. revealed that lncRNA-PRLB aggravated chemoresistance to paclitaxel in OCa via the RSF1/NF-κB pathway [11]. Teschendorff et al. also disclosed that lncRNA HOTAIR was closely associated with CBP resistance in OCa patients treated with CBP [12]. LINC01503 is a novel lncRNA that has been identified to be highly expressed in multiple human cancers and expedites cancer progression, including cancer derived from female reproductive system. For example, a report from Xie et al. revealed that LINC01503 exhibited high expression in squamous cell carcinoma and promoted cancer progression [13]. In addition, it was discovered that LINC01503 facilitated the malignant development of non-small-cell lung cancer by upregulating LASP1 as a sponge for miR-342-3p [14]. Furthermore, Peng et al. reported that LINC01503 was overexpressed in cervical cancer (CC) and contributed to CC tumorigenesis as an oncogene [15]. Additionally, a previous report from Li et al. identified LINC01503 as a lncRNA closely associated with OCa survival [16]. However, whether LINC01503 is involved in CBP resistance in OCa cells has never been studied.

Therefore, we intended to investigate whether LINC01503 could mediate CBP sensitivity in OCa via the miR-766-5p/PD-L1 axis, so as to find a new biomarker and develop novel strategies to attenuate CBP resistance in OCa.

## Materials and methods

### Cell culture and CBP treatment

Normal human ovarian epithelial cell line (IOSE-80) and human ovarian cancer cell lines (SKOV-3, OVCAR-10, CAOV-3, and OVCAR-3) were obtained from BeNa Culture Collection (Beijing, China) and cultured in an incubator (37 °C, 5% CO_2_) with RPMI1640 medium containing 10% FBS. SKOV-3 and OVCAR-10 cells were derived from ovarian serous cystadenocarcinoma and ovarian carcinoma, respectively. CAOV-3 and OVCAR-3 were derived from high-grade ovarian serous adenocarcinoma.

For the establishment of CBP-resistant OCa cell lines (OVCAR-3/CBP and CAOV-3/CBP), OVCAR-3 and CAOV-3 cells were continuously exposed to increasing concentrations of CBP (0–12 μM) over a period of 6 months. To maintain CBP resistance, OVCAR-3/CBP and CAOV-3/CBP cells were cultured in medium containing 6 μM CBP.

### Cell transfection

siRNA negative control (si-NC: 5ʹ-ACAAUCUUAUGUCGGGGCUCU-3ʹ), siRNA targeting LINC01503 (si-LINC01503: GCCTCTGACAAGTGTGTACCT), GATA1 (si-GATA1: 5ʹ-ACUAUGUCGGUCUCGAGAUCU-3ʹ), pcDNA3.1/LINC01503 (LINC01503), pcDNA3.1/GATA1 (GATA1), pcDNA3.1/PD-L1 (PD-L1), pcDNA3.1 (Vector), miR-766-5p mimics (miR-766-5p: 5ʹ-CGACUCCGAUUCUUGUUAGAG-3ʹ), and corresponding negative control (NC mimics: 5ʹ-AUGUGCUUAACCCUUUGGACG-3ʹ) were acquired from GenePharma (Shanghai, China). Lipofectamine 3000 (Invitrogen) was utilized to transfect OCa cells with si-NC, si-LINC01503, si-GATA1, LINC01503, GATA1, PD-L1, Vector, NC mimics, and miR-766-5p, respectively.

### Reverse transcription-quantitative (RT-q) PCR

With TRIzol reagent (Invitrogen), total RNA was isolated from cells and quantified with Nanodrop 2000 (Thermo Fisher Scientific) for RNA concentration. Next, total RNA was reversely transcribed into cDNA with 2 μg PrimeScript™ RT-PCR Kit (TaKaRa, Japan). Then, qPCR was performed on ABI 7300 Thermocycler (Thermo Fisher Scientific) with SYBR Premix Ex Taq kit (Thermo Fisher Scientific). Internal controls used were GAPDH and U6. The relative expressions were calculated by the 2^−ΔΔCt^ method. The sequences employed are as follows: GAPDH forward (F): 5ʹ-AACGGATTTGGTCGTATTGG-3ʹ and Reverse (R): 5ʹ-TTGATTTTGGAGGGATCTCG-3ʹ; GATA1 F: 5ʹ-CTTTCAGGTGTACCCATTGCT-3ʹ, and R: 5ʹ-TAGGTAGTGGCCTGTCCTGTC-3ʹ; LINC01503 F: 5ʹ-TGGTCATCTTTGGGTGGAGC-3ʹ, and R: 5ʹ-TGACCCAGTCTCCTGTCAGT-3ʹ; PD-L1 F: 5ʹ-GTCAGATTTTGTCCGTTCCACA-3ʹ, and R: 5ʹ-CATGGACTTGACGTAGCTGTT-3ʹ; U6 F: 5ʹ-CTCGCTTCGGCAGCACA-3ʹ, and R: 5ʹ-AACGCTTCACGAATTTGCGT-3ʹ; miR-766-5p F: 5′- TAAAATAGGAGTACTGTCTAA-3′, and R: 5′- ATTAGTAAATTGGCTGCTGCAG-3′.

### Western blot

Western blot assay was performed following the standard protocol. In brief, after total protein was isolated and quantified, the proteins were separated by SDS-PAGE (Solarbio, China) and transferred onto PVDF membranes (Pall Corporation, USA). Thereafter, the membranes were blocked in non-fat milk for 2 h, cultivated with primary antibodies against GATA1, PD-L1, E-cadherin, N-cadherin, Vimentin, Bcl-2, Bax, cleaved caspase-3 or GAPDH at 4 °C overnight, and then cultivated with HRP-conjugated secondary antibody (Sangon, China) for 2 h. The protein bands were analyzed under an enhanced chemiluminescence reagent (Beyotime).

### Cell Counting Kit-8 (CCK-8) assay

Half-maximal inhibitory concentration (IC_50_) was determined by CCK-8 to measure the sensitivity of OCa cells to CBP. Briefly, transfected OCa cells (1 × 10^4^/well) were transferred to 96-well plates and subject to different CBP concentrations (0–12 μM) for 72 h. Then, the cells were cultivated at 37 °C for 2 h after 10 μl CCK-8 reagent (Beotime, China) was added to each well. Finally, a microplate reader (Bio-Rad, USA) was employed to determine the absorbance at 450 nm, thereby assess cell viability.

### Colony formation assay

OCa cells (5 × 10^2^ cell/well) were seeded into a 6-well plate and exposed to 6 μM CBP for 48 h. Then, the cells were cultivated for 14 days. Thereafter, the colonies (> 50 cells) formed were counted and photographed with a light microscope (Olympus, Japan) after stained with 0.1% crystal violet.

### Transwell assay

1 × 10^5^ cells were transferred into the upper Transwell chamber. Meanwhile, DMEM with 10% FBS was supplemented to the lower Transwell chamber. With respect to migration detection, the membrane of the upper Transwell chamber is without Matrigel; concerning invasion detection, the membrane is precoated with Matrigel. After 24 h’ cultivation, the cells below the membrane were fixed and stained. The number of migrated or invaded cells was counted in five randomly selected visual fields with microscope.

### Flow cytometry

Transfected OVCAR-3/CBP and CAOV-3/CBP cells were put into 6-well plates (10^6^ cells/well) and cultivated with CBP for 48 h. Thereafter, cells were stained with annexin V-FITC/PI Apoptosis Detection Kit (KeyGEN Biotech, China). With FACScan flow cytometer (Becton Dickinson, USA) and CellQuest software (BD Biosciences, USA), the ratio of apoptotic cells was assessed.

### Dual-luciferase reporter assays

JASPAR database predicted the GATA1-binding sites in LINC01503 promoter. Next, OVCAR-3/CBP and CAOV-3/CBP cells were co-transfected with LINC01503 promoter sequences containing wildtype (WT) and mutant (MUT) binding sites to GATA1 together with GATA1 overexpression plasmid. To analyze the association between miR-766-5p and LINC01503/PD-L1, LINC01503 or PD-L1 3′UTR containing WT and MUT miR-766-5p binding sites were inserted into pmirGLO vectors. Thereafter, the established vectors were co-transfected in OVCAR-3/CBP and CAOV-3/CBP cells with NC mimics or miR-766-5p. After 48 h’ transfection, the dual-luciferase reporter assay system (Promega, USA) was conducted to measure the relative luciferase activity.

### Chromatin immunoprecipitation assay (ChIP) assay

EZ ChIP™ Chromatin Immunoprecipitation Kit (Millipore, USA) was employed for ChIP assay. Briefly, OVCAR-3/CBP and CAOV-3/CBP cells were fixed with PFA for 20 min’ crosslink and subject to sonication, thereby fragmenting DNA to 1000 bp in length. Next, DNA fragments were mixed with GATA1 or IgG antibody for immunoprecipitation overnight at 4 °C. Thereafter, the precipitated chromatin fragments were collected, purified, and analyzed via RT-qPCR.

### Statistical analysis

Every experiment was repeated 3 times. The data obtained were all presented as mean ± standard deviation. To perform a comparison between two groups or among multiple groups, Student’s t-test or one-way analysis of variance (ANOVA) was employed. GraphPad Prism 6.0 software was utilized for statistical analyses. *P* < 0.05 was deemed significant in statistics.

## Results

### LINC01503 overexpression is discovered in CBP-resistant OCa cells

**Fig. 1:**
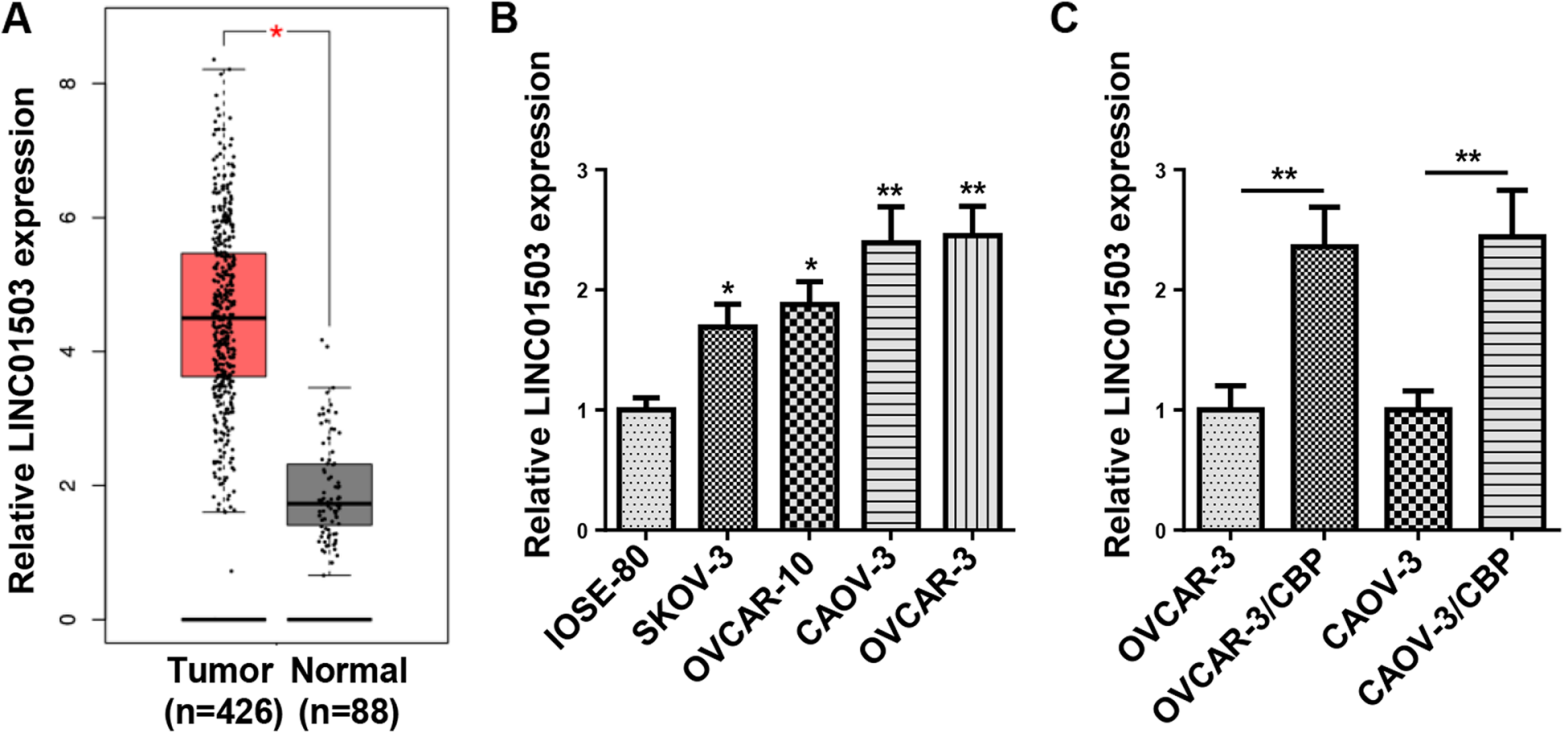
LINC01503 overexpression was discovered in CBP-resistant OCa cells. **A** The expression of LINC01503 in in OV samples (*n* = 426) and normal tissues (*n* = 88) was analyzed by TCGA database. **B** The expression of LINC01503 in OCa Cells (SKOV-3, OVCAR-10, CAOV-3, and OVCAR-3) and normal human ovarian epithelial cell line (IOSE-80) was detected by RT-qPCR. **C** RT-qPCR assay was used to detect the levels of LINC01503 in OVCAR-3/CBP and CAOV-3/CBP cells and their parental cells. (*n* = 3; *P < 0.05, **P < 0.01)

To identify the role of LINC01503 in OCa, The Cancer Genome Atlas (TCGA) database was employed to analyze LINC01503 abundance in Ovarian Serous Cystadenocarcinoma (OV). LINC01503 expression was significantly elevated in OV samples (*n* = 426), relative to normal tissues (*n* = 88) (Fig. 1A). Compared with the normal human ovarian epithelial cell line (IOSE-80), LINC01503 level was clearly upregulated in OCa Cells (SKOV-3, OVCAR-10, CAOV-3, and OVCAR-3), as indicated by RT-qPCR (Fig. 1B). As LINC01503 was most highly expressed in OVCAR-3 and CAOV-3 cell lines, the two cell lines were adopted for subsequent experiments. Then, CBP-resistant OCa cell lines (OVCAR-3/CBP and CAOV-3/CBP) were established to study the influence of LINC01503 on regulating biological behaviors and CBP resistance in OCa, and LINC01503 expression exhibited an increase in OVCAR-3/CBP and CAOV-3/CBP cells (Fig. 1C). The above results indicated that LINC01503 level was overexpressed in CBP-resistant OCa cells, suggesting LINC01503 could confer CBP resistance in OCa.

### LINC01503 depletion attenuates CBP resistance in CBP-resistant OCa cells

**Fig. 2:**
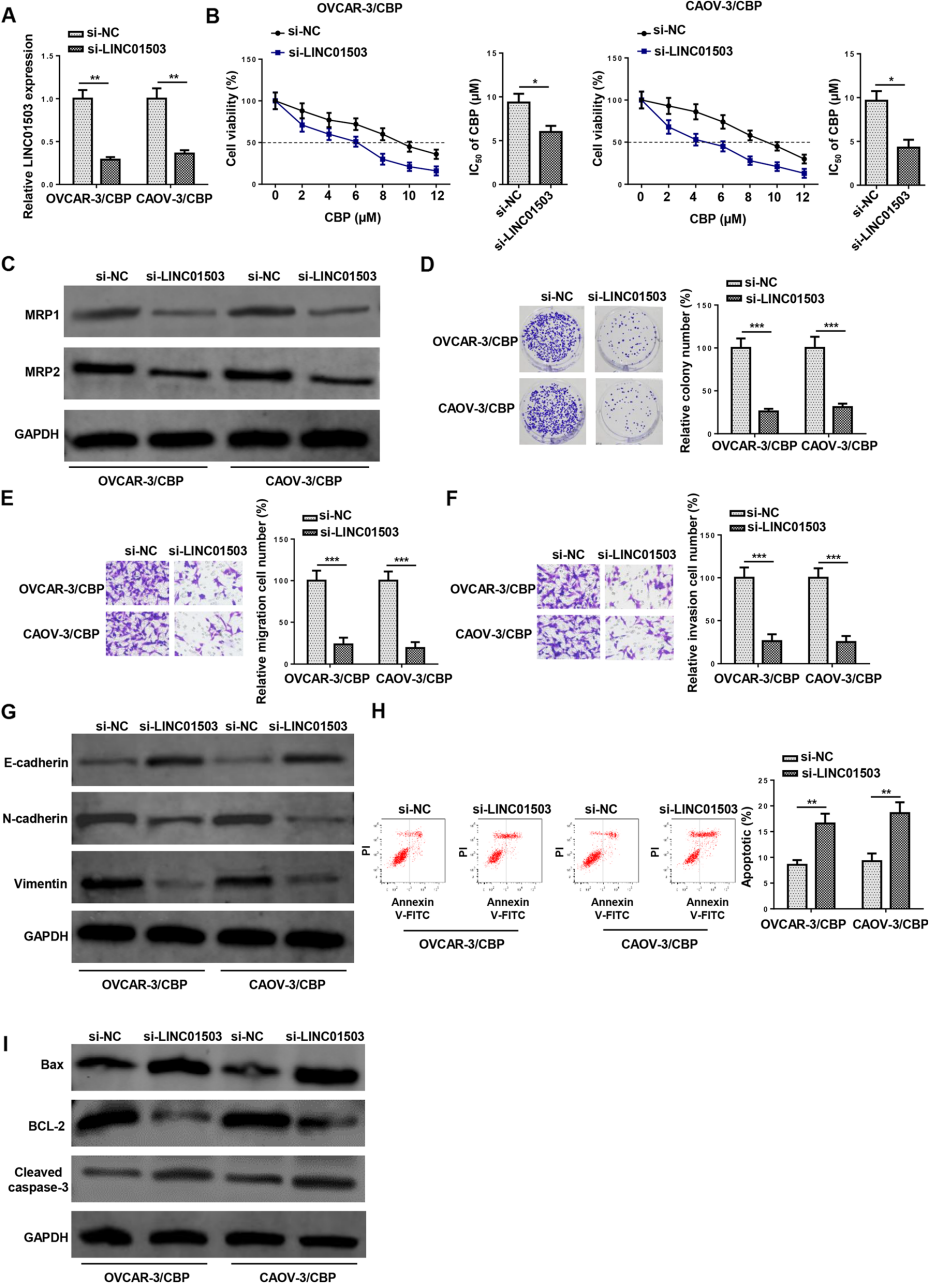
LINC01503 depletion attenuates CBP resistance in CBP-resistant OCa cells. **A** LINC01503 expression was measured by RT-qPCR in OVCAR-3/CBP and CAOV-3/CBP cells transfected with si-negative control (NC) or si-LINC01503. **B** CCK-8 assay was used to measure the IC50 value in CBP-resistant OCa cells transfected with si-NC or si-LINC01503. **D** Western blot showed the protein levels of MRP1 and MRP2 in CBP-resistant OCa cells transfected with si-NC or si-LINC01503. **E** Colony formation assay was used to detect the proliferation of CBP-resistant OCa cells transfected with si-NC or si-LINC01503. **F** and **G** Transwell assay was performed to detect the migration and invasion of CBP-resistant OCa cells transfected with si-NC or si-LINC01503. **H** Western blot showed the expression of E-cadherin, N-cadherin and Vimentin in CBP-resistant OCa cells. **I** Flow cytometry assay was performed to detect the apoptosis of CBP-resistant OCa cells transfected with si-NC or si-LINC01503. **J** Western blot showed the expression of Bax, Bcl-2, and cleaved caspase-3 in CBP-resistant OCa cells. (*n* = 3; *P < 0.05, **P < 0.01, ***P < 0.001)

To identify specific effects of LINC01503 on modulating the resistance to CBP in CBP-resistant OCa cells, LINC01503 was firstly silenced in OVCAR-3/CBP and CAOV-3/CBP cells (Fig. 2A). By CCK-8 assay, it was shown that LINC01503 inhibition significantly reduced IC_50_ in both OVCAR-3/CBP and CAOV-3/CBP cells (Fig. 2B). Furthermore, LINC01503 knockdown decreased the protein levels of chemoresistance-related proteins (MRP1 and MRP2) in OVCAR-3/CBP and CAOV-3/CBP cells (Fig. 2C). Colony formation assay indicated that LINC01503 depletion obviously repressed the proliferation of OVCAR-3/CBP and CAOV-3/CBP cells (Fig. 2D). In addition, it was also revealed by Transwell assay that LINC01503 silencing impaired the migrative and invasive capabilities of OVCAR-3/CBP and CAOV-3/CBP cells (Fig. 2E and F). Moreover, through detecting the expression of E-cadherin, N-cadherin and Vimentin, we found that silencing of LINC01503 inhibited the epithelial-mesenchymal transition process of OVCAR-3/CBP and CAOV-3/CBP cells (Fig. 2G). Furthermore, flow cytometry showed that the apoptotic rate of OVCAR-3/CBP and CAOV-3/CBP cells was largely increased after LINC01503 suppression (Fig. 2H), which was further confirmed by increased Bax and cleaved caspase-3 levels and decreased Bcl-2 level in both OVCAR-3/CBP and CAOV-3/CBP cells (Fig. 2I). Therefore, it was supposed that LINC01503 aggravated resistance to CBP in CBP-resistant OCa cells.

### GATA1 activates LINC01503 transcription in CBP-resistant OCa cells

**Fig. 3:**
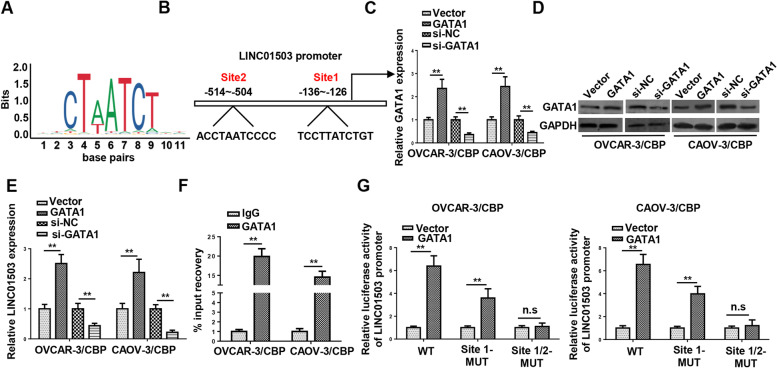
GATA1 activates LINC01503 transcription in CBP-resistant OCa cells. **A** and **B** The binding motif of GATA1 and LINC01503 promoter was presented according to the data from JASPAR and UCSC database. **C** and **D** RT-qPCR and western blotting were used to detect the mRNA and protein levels of GATA1 in CBP-resistant OCa cells transfected with GATA1 or si-GATA1. **E** The expression of LINC01503 was detected in OVCAR-3/CBP and CAOV-3/CBP cells by RT-qPCR. **F** ChIP assay revealed that there was a strong binding affinity of GATA1 and LINC01503 promoter on both 2 sites. **G** Luciferase reporter assay indicated the interaction between GATA1 and LINC01503 promoter. (*n* = 3; **P < 0.01)

Previous reports have shown that transcription factors are vital causes for dysregulated lncRNA expression in cancers [17–19]. To investigate the reason for upregulated LINC01503 expression in OVCAR-3/CBP and CAOV-3/CBP cells, JASPAR (http://jaspar.genereg.net/) was utilized to discover possible transcription factors of LINC01503. It was revealed that LINC01503 transcription was potentially mediated by GATA-binding protein 1 (GATA1), with 2 probable GATA1 binding sites for LINC01503 promoter (Fig. 3A and B). Thereafter, RT-qPCR and western blot confirmed that both mRNA and protein levels of GATA1 were increased after GATA1 overexpression and decreased after GATA1 inhibition in OVCAR-3/CBP and CAOV-3/CBP cells (Fig. 3C and D). Importantly, RT-qPCR showed that LINC01503 expression in OVCAR-3/CBP and CAOV-3/CBP cells was strengthened after GATA1 overexpression but declined by GATA1 inhibition, suggesting a positive relationship between GATA1 and LINC01503 in CBP-resistant OCa cells (Fig. 3E). In addition, the occupancy of GATA1 at the region of LINC01503 promoter was also demonstrated by ChIP assay (Fig. 3F). Moreover, results of luciferase reporter assay exhibited that the luciferase activity of LINC01503 promoter wildtype was obviously increased after the upregulation of GATA1; however, with mutation of the both two binding sites predicted, such an increase could be gradually neutralized and even completely offset in the end, indicating that GATA1 binds with LINC01503 promoter at both two sites (Fig. 3G). To sum up, GATA1 induced LINC01503 transcription in OCa cells resistant to CBP.

### LINC01503 is a sponge for miR-766-5p

**Fig. 4:**
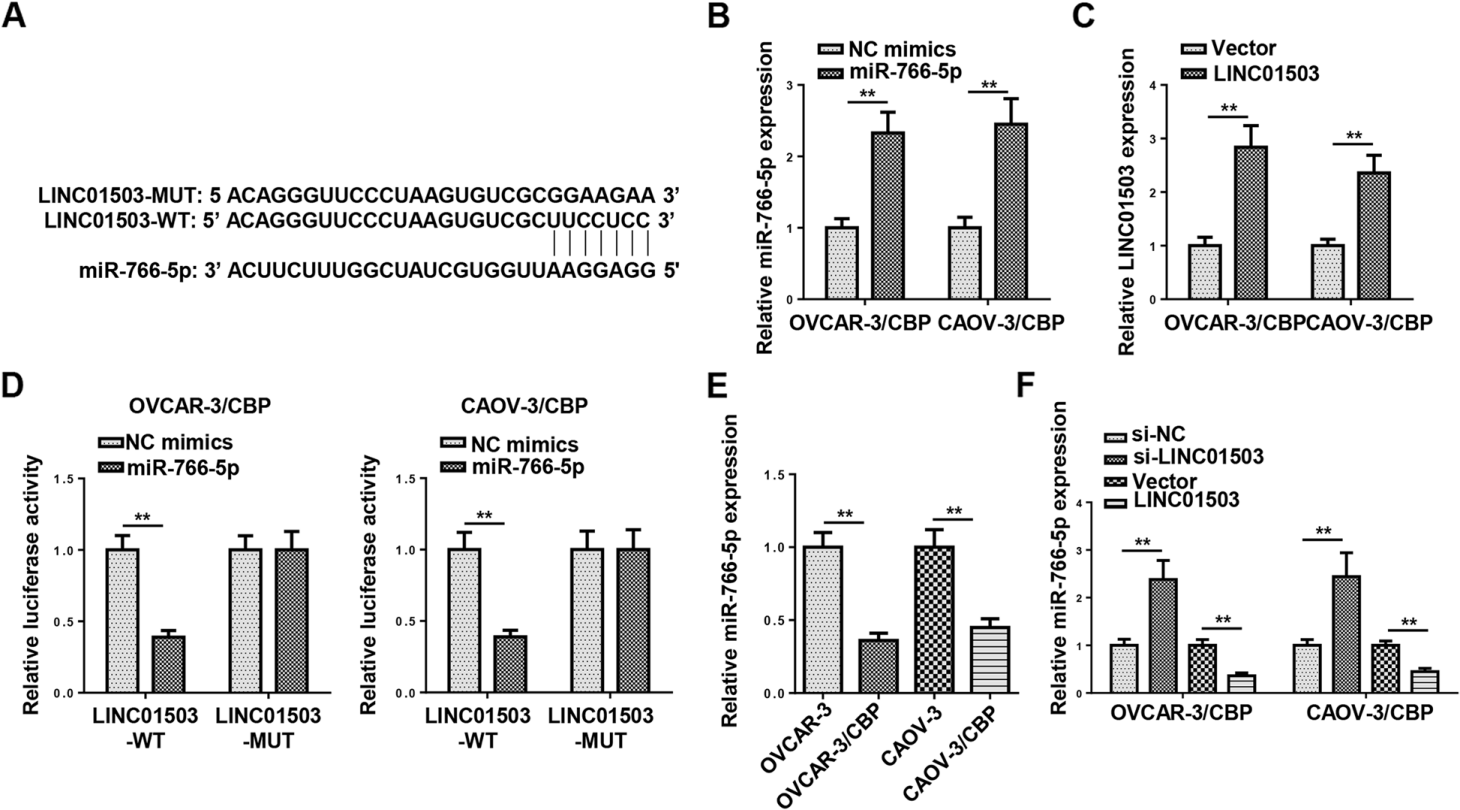
LINC01503 is a sponge for miR-766-5p. **A** The potential binding sites of miR-766-5p on LINC01503 was predicted by DIANA website. **B** and **C** Levels of miR-766-5p and LINC01503 were measured by RT-qPCR in OVCAR-3/CBP and CAOV-3/CBP cells. **D** Luciferase activities of wild-type or mutant LINC01503 in CBP-resistant OCa cells transfected with NC mimics or miR-766-5p mimics were detected by luciferase reporter assay. **E** The expression levels of miR-766-5p in CBP-resistant and sensitive OCa cell lines were measured by RT-qPCR. **F** The expression levels of miR-766-5p in OVCAR-3/CBP and CAOV-3/CBP cells transfected with Vector, LINC01503, si-NC, and si-LINC01503 were determined by RT-qPCR. (*n* = 3; **P < 0.01)

DIANA-LncBase v.2 provides an extensive amount of predicted miRNA targets on the largest available set of human lncRNAs [20]. To further explore the mechanism of LINC01503-regulated CBP resistance in OCa, DIANA website predicted miR-766-5p as a downstream target of LINC01503 with high score. The latent binding sites between miR-766-5p and LINC01503 were as shown in Fig. 4A. Transfection efficiency of miR-766-5p and LINC01503 overexpression was verified by RT-qPCR assay (Fig. 4B and C). Luciferase reporter assay exhibited that miR-766-5p overexpression significantly reduced luciferase activity of LINC01503-WT rather than LINC01503-MUT in OVCAR-3/CBP and CAOV-3/CBP cells (Fig. 4D). Besides, miR-766-5p expression was remarkably lower in OVCAR-3/CBP and CAOV-3/CBP cells than in their parental cell lines (OVCAR-3 and CAOV-3) (Fig. 4E). Furthermore, LINC01503 inhibition upregulated miR-766-5p expression, while the miR-766-5p level was reduced by LINC01503 overexpression in OVCAR-3/CBP and CAOV-3/CBP cells (Fig. 4F). In sum, miR-766-5p was lowly expressed and negatively regulated by LINC01503 in CBP-resistant OCa cells.

### LINC01503 contributes to CBP resistance of OCa cells via miR-766-5p

**Fig. 5:**
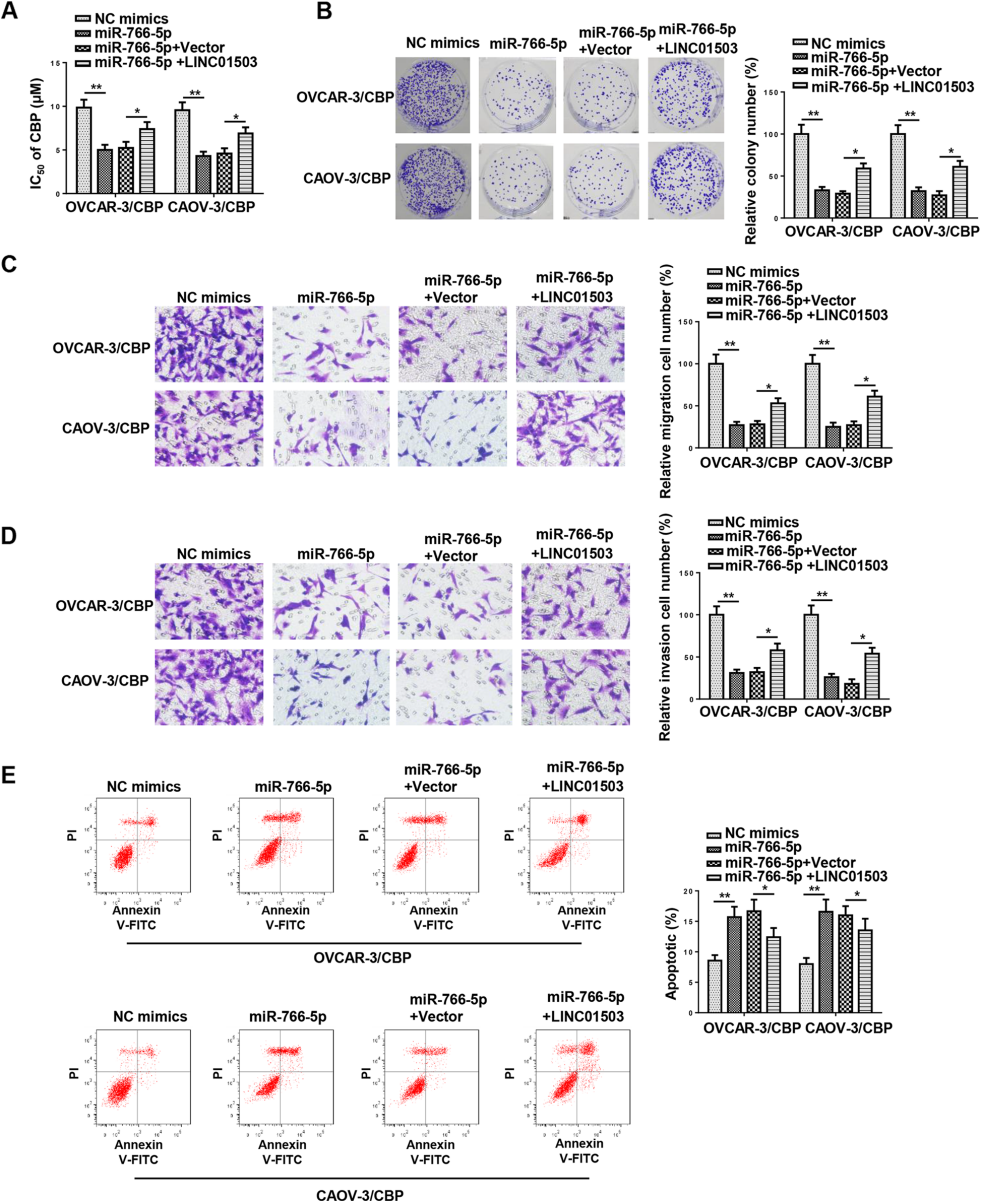
LINC01503 contributes to CBP resistance of OCa cells via miR-766-5p. OVCAR-3/CBP and CAOV-3/CBP cells were transfected with NC mimics, miR-766-5p mimics, miR-766-5p mimics + Vector, and miR-766-5p mimics + LINC01503. **A** and **B** CCK-8 and colony formation assays were used to detect the proliferation of CBP-resistant OCa cells. **C** and **D** Transwell assay was performed to detect the migration and invasion of CBP-resistant OCa cells. **E** Flow cytometry assay was performed to detect the apoptosis of CBP-resistant OCa cells. (*n* = 3; *P < 0.05, **P < 0.01)

To further explore whether LINC01503 regulated CBP resistance of OCa through miR-766-5p, functional assays were performed. Firstly, OVCAR-3/CBP and CAOV-3/CBP cells were transfected with NC mimics, miR-766-5p, miR-766-5p + Vector, and miR-766-5p mimics + LINC01503. By CCK-8 and colony formation assays, it was discovered that miR-766-5p overexpression obviously reduced IC_50_ and impaired cell proliferation in OVCAR-3/CBP and CAOV-3/CBP cells, and such suppression was eliminated by LINC01503 overexpression (Fig. 5A and B). Besides, miR-766-5p upregulation restrained the migrative and invasive abilities of OVCAR-3/CBP and CAOV-3/CBP cells, and such repression could be abolished after LINC01503 addition (Fig. 5C and D). Moreover, the accelerated OVCAR-3/CBP and CAOV-3/CBP cell apoptosis induced by elevated miR-766-5p expression was also abrogated by the addition of LINC01503 abundance (Fig. 5E). Taken together, it was disclosed that LINC01503 contributed to CBP resistance in OCa via regulating miR-766-5p.

### PD-L1 is a target of miR-766-5p and can be regulated by LINC01503 via miR-766-5p

**Fig. 6:**
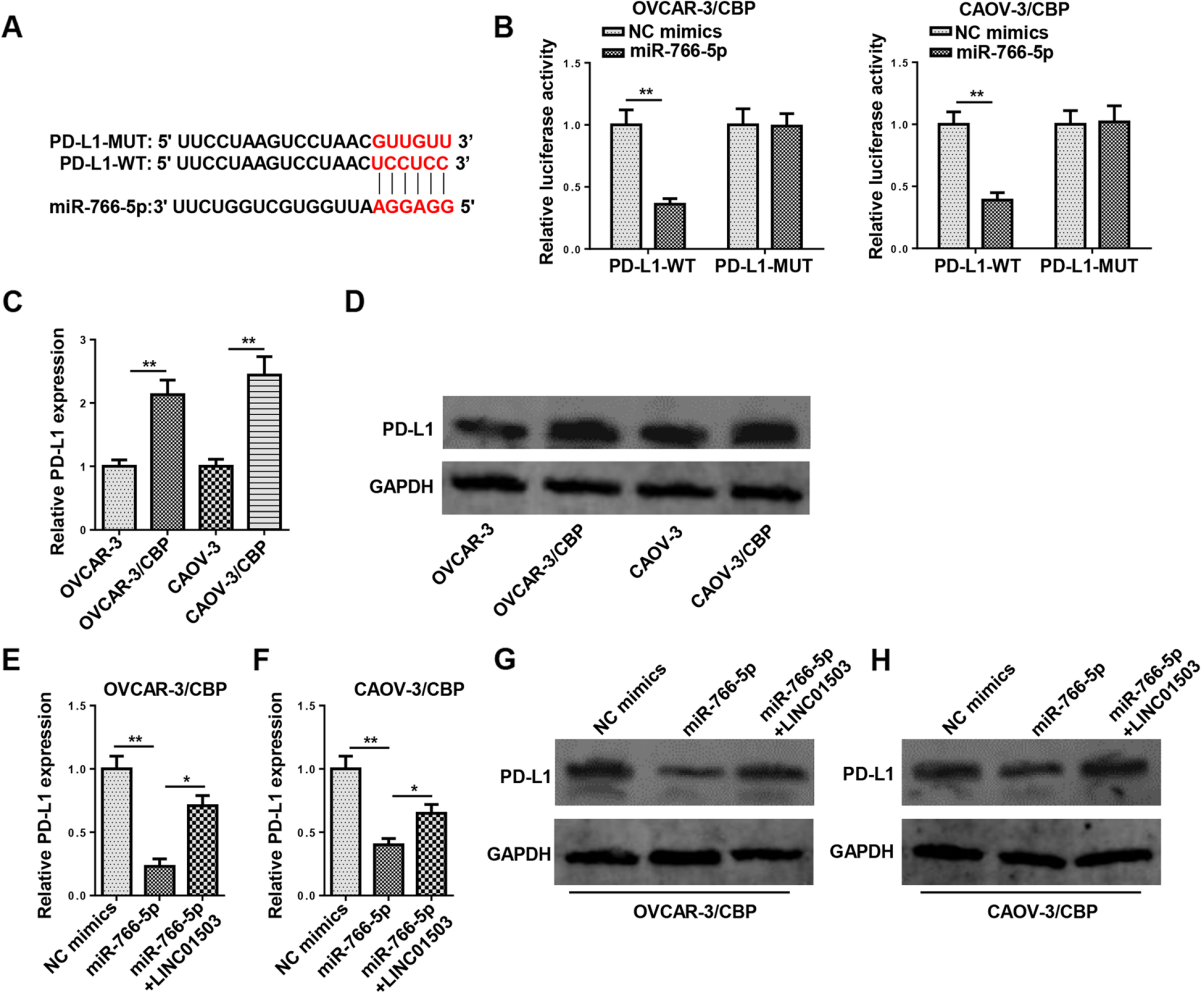
PD-L1 is a target of miR-766-5p and can be regulated by LINC01503 via miR-766-5p. **A** Putative binding regions of miR-766-5p in 3′UTR of PD-L1 was predicted by StarBase. **B** Luciferase activities of wild-type or mutant PD-L1 in OVCAR-3/CBP and CAOV-3/CBP cells transfected with NC mimics or miR-766-5p mimics were determined by luciferase reporter assay. **C** and **D** The mRNA and protein levels of PD-L1 in CBP-resistant OCa cell lines and parental cell lines were detected by RT-qPCR and Western blotting assays. **E**–**H** The mRNA and protein levels of PD-L1 were measured by RT-qPCR and western blotting in OVCAR-3/CBP and CAOV-3/CBP cells. (*n* = 3; *P < 0.05, **P < 0.01)

To further investigate the downstream mechanism of LINC01503/miR-766-5p axis, StarBase website was employed for bioinformatics analysis. Among the potential targets of miR-766-5p, PD-L1 (CD274), an inhibitory immune receptor, has been identified as a key target of immunotherapy for cancers by various studies [21]. Moreover, high PD-L1 level is a predictive biomarker and closely related to platinum-based chemoresistance in a variety of human cancers, including OCa [22–24]. Therefore, PD-L1 was chosen as the downstream target of LINC01503/miR-766-5p axis, and the 3′-UTR of PD-L1 displayed complementary points for miR-766-5p (Fig. 6A). Then, dual-luciferase reporter assay showed that the addition of miR-766-5p distinctly decreased the luciferase activity of PD-L1-WT in OCa cells, relative to PD-L1-MUT (Fig. 6B). In addition, PD-L1 mRNA and protein levels were remarkably increased in CBP-resistant OCa cells, compared with the parental OCa cells (Fig. 6C and D). It was revealed that there was a significant decrease of PD-L1 expression in OVCAR-3/CBP and CAOV-3/CBP cells expressing miR-766-5p overexpression, while such a decline could be reversed by LINC01503 upregulation (Fig. 6E–H). It is evidenced by our data that PD-L1 was upregulated by LINC01503 via miR-766-5p.

### LINC01503 promotes CBP resistance in OCa cells by regulating PD-L1

**Fig. 7:**
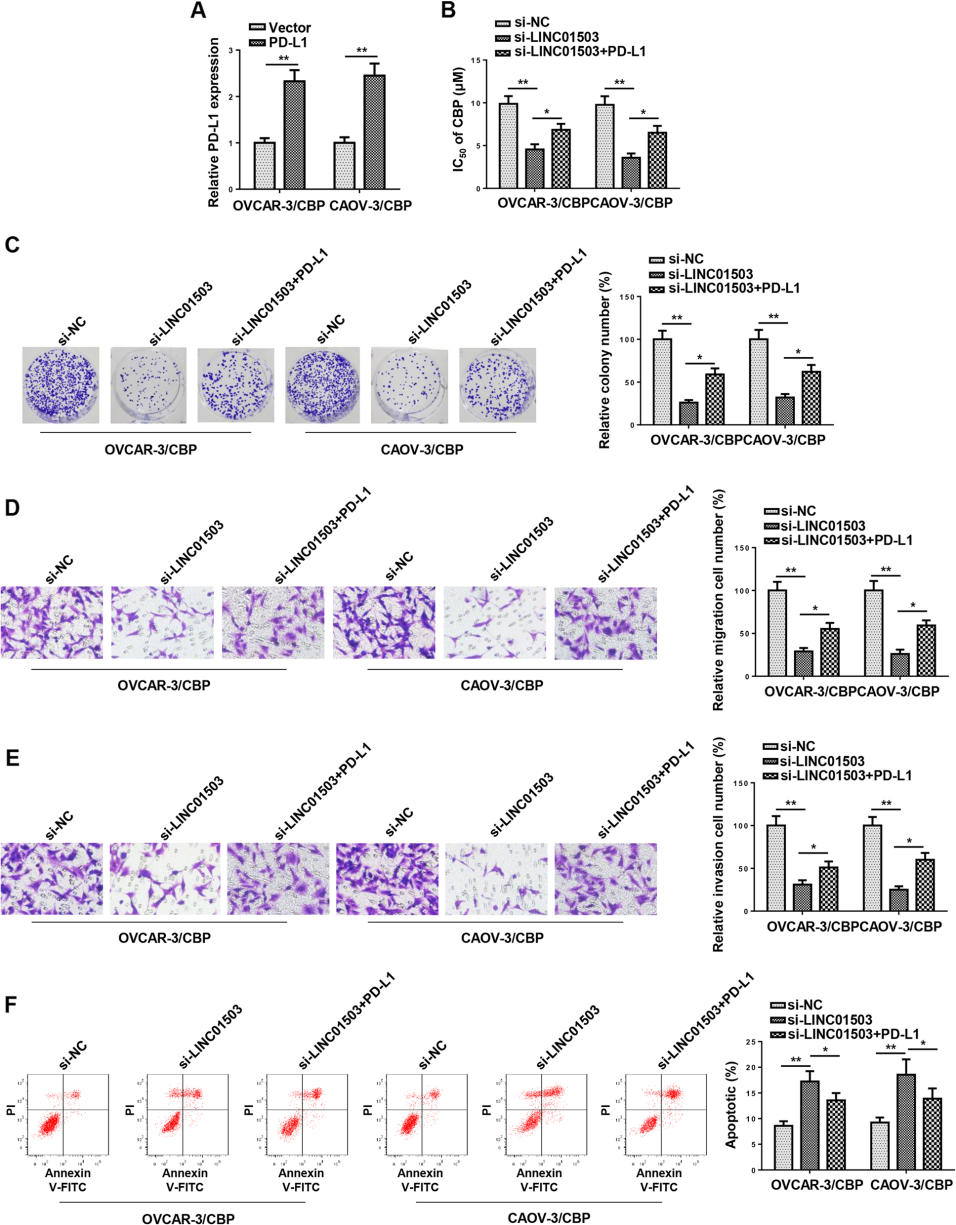
LINC01503 promotes CBP resistance in OCa cells by regulating PD-L1. **A** PD-L1 expression was measured by RT-qPCR in OVCAR-3/CBP and CAOV-3/CBP cells transfected with Vector or PD-L1. **B** and **C** CCK-8 and colony formation assays were used to detect the proliferation of OVCAR-3/CBP and CAOV-3/CBP cells. **D**-**F** Transwell and TUNEL assays were performed to measure the migration, invasion and apoptosis of OVCAR-3/CBP and CAOV-3/CBP cells. (*n* = 3; *P < 0.05, **P < 0.01)

To inquire whether PD-L1 was a downstream regulator of LINC01503/miR-766-5p axis to regulate CBP resistance in OCa cells, PD-L1 was firstly overexpressed in OVCAR-3/CBP and CAOV-3/CBP cells (Fig. 7A). Then, a series of rescue experiments were performed. It was indicated that LINC01503 blocking reduced CBP IC_50_, cell viability, migration, and invasion but facilitated apoptosis in OVCAR-3/CBP and CAOV-3/CBP cells, whereas PD-L1 overexpression neutralized these effects (Fig. 7B-F). These results indicated that LINC01503 promoted CBP resistance in OCa cells by elevating PD-L1 expression.

## Discussion

Ovarian Carcinoma (OCa) is a fetal malignancy derived from female reproductive system. CBP is a platinum complex widely used to treat human cancers, including OCa [25–27]. In recent years, an increasing number of researches have proven that lncRNAs exert significant effects in regulating chemoresistance of human cancers, including OCa [28–30].

As demonstrated by accumulated evidence, LINC01503 aggravates the progression of several malignancies, such as nasopharyngeal carcinoma [31], cholangiocarcinoma [32], and hepatocellular carcinoma [33]. According to an investigation performed with TCGA, LINC01503 was highly expressed in OV tissues, compared with corresponding normal tissues. LINC01503 abundance was also elevated in OCa cell lines, relative to normal human ovarian epithelial cell lines. Moreover, LINC01503 expression was also largely increased in CBP-resistant OCa cell lines, in comparison to corresponding parental OCa cell lines. Furthermore, it was also demonstrated that LINC01503 knockdown apparently declined CBP IC_50_, restrained cell proliferation, and impaired migration and invasion in CBP-resistant OCa cells; however, LINC01503 depletion also accelerated apoptosis in CBP-resistant OCa cells, indicating LINC01503 enhanced CBP resistance in OCa cells.

GATA1 has been identified as a key transcription factor participating in the Triple-Negative Breast Cancer by regulating the expression of lncRNA HNF1A-AS1 [34]. A previous study by Liu et al. also demonstrated that GATA1 was upregulated in OCa and accelerated OCa development by regulating JAG1 level as a transcription factor [35]. Here, we predicted GATA1 as a transcription factor of LINC01503. Through mechanism assays, it was discovered that GATA1 interacted with LINC01503 promoter and promoted LINC01503 expression. It was demonstrated that LINC01503 transcription could be activated by GATA1 in CBP-resistant OCa cells.

Herein, we identified miR-766-5p as the downstream miRNA of LINC01503 in OCa. It has been demonstrated that miR-766-5p is lowly regulated in lung cancer and alleviates lung cancer tumorigenesis [36, 37]. Zhao et al. discovered that miR-766 restrained the development of papillary thyroid cancer by PI3K/Akt pathway via regulating IRS2 [38]. These reports indicated that miR-766-5p could be a tumor suppressor in several human cancers. In consistent with previous studies, we found a downregulation of miR-766-5p in OCa cells. Then, we confirmed PD-L1 as a downstream target of miR-766-5p in CBP-resistant OCa cells. It has been firmly demonstrated by a number of studies that PD-L1 exhibited dysregulation and chemoresistance-promoting role in human cancers. For example, Shen et al. reported that PD-L1 promoted cisplatin resistance in head and neck squamous cell carcinoma by synergizing with MRN [23]. Wang et al. demonstrated that PD-L1 upregulation enhanced paclitaxel resistance in glioma via interaction with miR-34a [39]. Moreover, Zuo et al. also demonstrated that PD-L1 was highly expressed and conferred chemoresistance in OCa cells [40]. In our research, we discovered a remarkable increase of PD-L1 level in CBP-resistant OCa cells. Besides, PD-L1 was positively regulated by LINC01503 and negatively modulated by miR-766-5p. Furthermore, rescue experiments demonstrated that PD-L1 overexpression could neutralize the suppressive effect of LINC01503 knockdown on CBP resistance in OCa cells.

To sum up, our study, for the first time, investigated the molecular mechanism of the GATA1-activated LINC01503/miR-766-5p/PD-L1 network in regulating resistance of OCa cells to carboplatin. We also demonstrated that LINC01503 upregulation induced by GATA1 exacerbated resistance to carboplatin in OCa cells via modulating the miR-766-5p/PD-L1 axis. Our findings provide a novel insight into developing more targeted and customized chemotherapeutic strategies for OCa treatment.

## Data Availability

The datasets used and/or analyzed during the current study are available from the corresponding author on reasonable request.
